# Lure specificity, phenology, and damage caused by *Epiphyas* moths (Lepidoptera: Tortricidae) in Western Australian apple orchards

**DOI:** 10.1093/jee/toae162

**Published:** 2024-07-24

**Authors:** Maryam Yazdani, Elliot Howse, Wee Tek Tay, Helen Spafford, Rieks D van Klinken

**Affiliations:** Commonwealth Scientific and Industrial Research Organisation (CSIRO ), Health & Biosecurity, Brisbane, QLD 4001, Australia; Department of Primary Industries and Regional Development (DPIRD), South Perth, WA, Australia; Commonwealth Scientific and Industrial Research Organisation (CSIRO ), Health & Biosecurity, Canberra, ACT 2601, Australia; Department of Primary Industries and Regional Development (DPIRD), South Perth, WA, Australia; Commonwealth Scientific and Industrial Research Organisation (CSIRO ), Health & Biosecurity, Brisbane, QLD 4001, Australia

**Keywords:** light brown apple moth, horticulture, pheromone lure, tortricid moth, population dynamics

## Abstract

Multiple *Epiphyas* species inhabit southwestern Western Australia, including Light Brown Apple Moth (LBAM) *Epiphyas postvittana* (Walker) (Lepidoptera: Tortricidae), a globally significant, polyphagous pest. This study evaluated the efficacy and specificity of lures designed for 3 *Epiphyas* species: *E. postvittana*, *Epiphyas pulla* (Turner), and the undescribed *Epiphyas sp. (1)* (Common). Additionally, the study sought to determine the presence and distribution of *Epiphyas* species in 3 significant apple-growing localities. Trapping, together with partial sequencing of the mitochondrial COI gene, found LBAM to be restricted to the Perth Hills and *E. pulla*, to apple orchards near Manjimup and Pemberton. This geographic disjunction remains unexplained. *Epiphyas sp. (1)* was not recorded despite using a specifically designed lure. The *E. pulla* and LBAM traps demonstrated superior efficacy in capturing their target species, while the catch in *Epiphyas sp. (1)* traps did not significantly differ between the 2. Both *E. pulla* and LBAM exhibited peak abundance from late spring to the end of summer (October–February), with variations in timing and peak catch of male moths across species, locations, and years. Surveys conducted in April during the harvest period (February–May), when moth traps caught an average of 1–1.8 moths/trap/week, found no *Epiphyas* larvae or damage on 140,400 mature apples or on 26,000 leaves. While *E. pulla* and LBAM traps effectively monitor their target moths, genetic identification of trap catch would be necessary if they co-occurred. Encouragingly, the results indicate that both species become relatively rare as harvest season approaches, and neither inflicts significant damage to mature apples under existing management.

## Introduction

*Epiphyas* is the largest genus within the Australian Tortricinae moths (leaf-rollers) (Hitchcock 2012). In southwestern Western Australia, 2 species have been reported as pests of apples (*Malus domestica*). Adults of an additional undescribed species have been collected from cultivated environments in Western Australia and South Australia (Hitchcock 2012). *Epiphyas postvittana* (Walker) (Lepidoptera: Tortricidae), commonly known as Light Brown Apple Moth (LBAM), can be a significant pest of apples and other horticultural crops. Native to eastern Australia, it has naturalized in Western Australia and has also been established around the world, including in New Zealand, Hawaii, England, and California (Danthanarayana 1975, Geier and Springett 1976, Wearing et al. 1991, Suckling and Brockerhoff 2010), probably following the import of nursery stock (Suckling and Brockerhoff 2010). *Epiphyas pulla* (Turner) (Lepidoptera: Tortricidae) is currently restricted to southwestern Western Australia, where it is considered endemic (Hitchcock 2012). Historically, this has caused severe losses to pome-fruit crops around Manjimup (Geier and Springer 1976). The third undescribed species, labeled as “*Epiphyas sp. (1)* (Hitchcock 2012),” occasionally had males trapped in Western Australia with the *E. pulla* pheromone lure blend. This species has been found in cultivated crops in Western Australia and South Australia. However, there are no reports of it feeding on apple trees, and little is known about its relative importance to the apple industry in Western Australia. (Hitchcock 2012).

Like many members of the Archipini tribe, early LBAM instars primarily feed on the undersides of leaves while residing within a protective silk chamber. As they progress to later instars, their behavior shifts toward folding individual leaves, constructing nests from several interconnected leaves, or attaching leaves to fruit surfaces for feeding. Larvae forage on the leaves, buds, flowers, and fruits of their host plants (Brown et al. 2010). The most significant economic impact arises from larvae feeding on the surface of fruits concealed within their webbed leaf shelters. Surface feeding results in scarring and provides opportunities for secondary infections, diseases, and rots (Bailey et al. 1996, USDA-APHIS-PPQ 2008).

*Epiphyas postvittana* is highly polyphagous, with over 500 known hosts spanning 121 families and 363 genera (Danthanarayana 1975). This includes many native hosts in Australia (Clark 1970, Danthanarayana 1975). Its natural hosts are thought to be evergreen acacia species (Suckling and Brockerhoff 2010). Larvae tend to show a preference for herbaceous plants over woody ones (Danthanarayana et al. 1995, Suckling and Brockerhoff 2010). Surveys suggest they may favor understory plants that occur below 1.5 m and small, dense plants (Geier and Briese 1980a). *Epiphyas postvittana* can cause significant damage to apples, citrus fruits, and grapes (Danthanarayana 1975, Buchanan 1977). Wearing et al. (1991) reported up to 70% damage to fruit crops in Australia and New Zealand when left unmanaged. The presence of alternative hosts could allow persistence in orchards. For instance, in Canberra (Australian Capital Territory (ACT)), immature apple trees supported the LBAM population from late spring to autumn. In winter and early spring, clover and broadleaf weeds were the main hosts (Geier and Briese 1980a). Evidence also suggested that maturing apple trees “grow out” of LBAM infestations (Geier and Briese 1980a).

*Epiphyas pulla* has been recorded from hosts in the families Rosaceae and Actinidiaceae (Hitchcock 2012) but has only been reported as a pest of apples (Geier and Springer 1976). *Epiphyas pulla* is poorly studied, but it can be found in similar crops and habitats as LBAM (Hitchcock 2012). *Epiphyas sp. (1)* has been reported on several native plant species and on olive (*Olea europaea*) and bay trees (*Laurus nobilis*) (Hitchcock 2012), but there are no reports of it being a horticultural pest.

Synthetic sex pheromones are routinely used in traps to monitor adult flight, abundance, phenology and can attribute a biofix date for degree day phenology modellings (Way and van Emden 2000, Baker 2008). Naturally produced sex pheromones can be highly species-specific and operate in specific biological contexts to attract members of the opposite sex for mating (Phillips et al. 2000). Comeau and Roelofs (1973) investigated attraction specificity in Tortricidae using synthetic pheromones under laboratory and field conditions. They demonstrated that species are highly specific in their attraction to their own pheromones. This can occur even when they share a common primary attractant due to differences in seasonal cycles, circadian rhythms, and secondary chemicals emitted alongside primary pheromones. These secondary chemicals vary in composition and concentration among species, enhancing the specificity observed in attraction behaviors (Comeau and Roelofs, 1973).

Two primary components were found in the sex pheromone glands of female *E. postvittana*, E11-14:Ac and E9E11-14:Ac (Bellas et al. 1983). The range of E9E11-14:Ac varied naturally in sex pheromone glands from 100:2.2 to 100:11.4, and different ratios of binary blend had limited effect on trap catch (Muggleston and Foster, 1989). In another study, male LBAM exhibited responses to ratios for these 2 components ranging from 10:1 to 50:1, although the optimal attraction ratio for males was suggested to be 100:5 (Bellas et al. 1983). A synthetic pheromone lure has been developed for LBAM using these 2 components and is widely used internationally to monitor this moth (El-Sayed et al. 2011, Zielonka et al. 2021). The sex pheromone of *Epiphyas sp. (1),* sourced from the moths captured in a glasshouse in Perth, was analyzed and found to contain the same bioactive components as LBAM, but in the ratio of 10:1 (Manning et al. 2009) as cited in Hitchcock (2012). A commercial lure is also available for *E. pulla* which includes an additional 2 components (n-14:Ac and n-16:Ac) (Hitchcock 2012).

We conducted field trials to test the specificity of lures developed for each of the 3 species and used the most effective lures to determine the distribution and phenology of *Epiphyas* species present in apple orchards in southwestern Western Australia. The 3 species are very difficult to distinguish morphologically (Hitchcock, 2012). Therefore, we used DNA sequencing to identify species caught in the lure specificity trial. Traps can be used to monitor adult moth activity, but that does not necessarily correlate with levels of damage to apple trees or apples. Therefore, field inspections were conducted in apple orchards to detect the presence of any life stages of *Epiphyas* moths on apple trees. Additionally, fruit inspections were carried out in apple orchards where *Epiphyas* moths were captured in traps to determine whether there is any correlation between fruit damage and catch in traps.

## Materials and Methods

### Study System and Region

Research was conducted in the 2020–2021 and 2021–2022 apple seasons across 3 apple-growing regions in southwestern Western Australia: Perth Hills (approximately 20 km east of Perth; −32.034995, 116.132179), Manjimup (approximately 257 km south of Perth; −34.243236, 116.145442) and Pemberton (approximately 278 km south of Perth; −34.445604, 116.033275). The region has a Mediterranean climate featuring hot, dry summers (December to February) and cool, wet winters (June to August).

The Bureau of Meteorology (BOM) data for the years 2018–2022 reveals distinct climatic characteristics in 3 locations. Perth Hills (BOM station—9913 Roleystone) had the lowest annual rainfall (548 mm), experienced the hottest summers (average daily maximum temperatures 30 °C), and lowest humidity (32.7%). Summers were dry (52.2 mm of rainfall). Manjimup (BOM Station—9573 Manjimup) had intermediate annual rainfall (628 mm), summer temperatures (27.8 °C) and humidity (38.1%), and the same summer rainfall (52.1 mm). Pemberton (BOM—9904 Walters Farm) had the highest annual rainfall (860 mm), coolest summers (25.9 °C), highest humidity (49%) and highest summer rainfall (70.6 mm).

Perth Hills has agricultural and peri-urban land uses, while Manjimup and Pemberton are both rural towns that are surrounded predominantly by horticulture, pasture, and forestry.

### Moth Traps and Lures Used in This Study

Three pheromone lures were evaluated during this study, and their composition and source are described in Table 1. The components of LBAM lures are widely employed globally especially in mating disruption, serving as the predominant field-control tactic (Suckling and Brockerhoff 2010). In contrast, the *E. pulla* lure was specifically developed for research purposes within Australia. *Epiphyas pulla* and *Epiphyas sp. (1)* lures are not commercially available.

**Table 1.** The relative composition of active components and the supplier for each of the 3 tested lures

Component	Tested lure (ratio in 1 mg plugs)		
	LBAM (20:1)	*E. pulla* (10:16:10:1)	*Epiphyas sp. (1)* (10:1)
E11-14:Ac	95%	27%	91%
E9,E11-14:Ac	5%	43%	9%
n-14:Ac	–	27%	–
n-16:Ac	–	3%	–
**Lure supplier**	Bugs for Bugs, Toowoomba, Queensland	Richard Vickers, (formerly with CSIRO Entomology’s Pheromone Research Group) ACT (R. Vickers, pers. comm. 2009 cited by Hitchcock 2012)	Plant and Food Research, Auckland, New Zealand (Lee-Anne Manning, Vanessa Mitchell, and Max Suckling, P&F-NZ, Lincoln, 2009 unpublished results, cited in Hitchcock 2012)

Traps consisted of a Desire red delta (UPL-Ltd, Auckland, New Zealand), a sticky base (Bugs for Bugs, Toowoomba, Queensland), and a rubber-septum impregnated with 1mg of the target species’ pheromone mixture (Table 1) placed in the center of each sticky base using a long pair of tweezers.

### Lure Specificity Trial (2021–2022)

A lure specificity trial was conducted with weekly trapping from October to December (2021–2022) in the Perth Hills (a 20 ha mixed apple/stone fruit orchard) and in apple orchards from December to the end of January in Manjimup (12 ha) and Pemberton (22 ha). The trial timing aligned with the predicted peak abundance of moths in each locality, as indicated by historical data. All *Epiphyas* specimens were identified as species using the DNA barcoding approach. This data was also used to supplement phenology data (see below).

Replicates of pairs of LBAM and *E. pulla* pheromone trap sets (*n* = 10) were established in the Perth Hills orchard. In addition, trios of LBAM, *E. pulla*, and *Epiphyas Sp.* (1) pheromone traps were replicated in the Manjimup (*n* = 6 replicates) and Pemberton (*n* = 10) orchards.

The sets of traps were evenly distributed throughout the orchards, and the placement of lures was randomized. Traps were placed at a minimum distance of 50 m apart to avoid pheromone interference (El-Sayed et al. 2011), and the lure type was randomized for each trap location. Traps were placed either at the middle or edge of apple rows along the eastern side of trees, 1.8 m from the ground.

All traps were checked weekly, the number of moths in each trap was recorded and the adhesive base replaced. Pheromone lures were replaced monthly. Adhesive bases with trapped moths were stored in a freezer at −18 °C until samples were processed.

To prepare the samples for genetic identification, the heads of moths that were morphologically identified as *Epiphyas* species were carefully removed from their bodies using fine forceps. Heads were then placed in 15 ml Nunc conical centrifuge tubes containing ≥5 ml of 100% undenatured ethanol. Ethanol was replaced every 24 hours until ethanol had been changed out 4 times. Sample tubes were kept in the freezer at −18 °C until used for DNA extraction.

### Monitoring LBAM and *E. pulla* Phenology (2020–2021)

Additional trapping was conducted in 2020–2021 to monitor the seasonal abundance of LBAM and *E. pulla* across apple orchards in the Perth Hills (*n* = 4 paired traps), Manjimup (*n* = 16), and Pemberton (*n* = 4). Traps were placed in the field from bud burst (October) through to the well after harvest completion (August). All traps were placed on the eastern side of apple trees at a height of 1.8 m, with one pair of LBAM and *E. pulla* traps placed in either the middle or edge of rows and at least 25 m apart.

Traps underwent monthly servicing, including the monthly replacement of lures. The adhesive base of the traps was visually assessed at the time of servicing for *Epiphyas* moths. If moths were observed, the adhesive base was substituted with a fresh one. The removed adhesive base was labeled and inserted into a plastic sleeve. *Epiphyas* moths were subsequently identified morphologically at the genus level and counted. This was based on the distinctive male fore wing pattern of the Archipini group, encompassing a basal patch, median transverse fascia, and costal triangle (Common 1963).

### DNA Extraction and Specimen Identification

The QIAGEN DNeasy 96 Blood & Tissue Kit was used for DNA extraction, following the protocol provided by the manufacturer (Hilden, Germany). Before starting the lysis step, the preserved head samples were transferred onto Kimwipes paper tissues to dry out the excess ethanol. Briefly, for sample lysis, 180 µl of ATL buffer mixed with 20 µl of Proteinase K was added to individual heads and incubated at 56 °C for 16–24 h (overnight). After incubation, 4 µl of RNase A was added to each sample and incubated at room temperature for 2 min. A specified volume of Buffer AL mixed with an equal volume of absolute ethanol was added to each sample as per the manufacturer’s instructions and centrifuged to bind DNA to the membrane, followed by 2 washes prior to eluting the DNA using the Qiagen Elution Buffer (100 µl for each sample).

For PCR amplification, a set of mitochondrial cytochrome oxidase subunit I (mtCOI) primers was developed for *Epiphyas* species by identifying the conserved terminal mtCOI gene regions shared between different species. To design the universal *Epiphyas* COI primers (*Epiphyas*_COIF: 5ʹ-ATTTGAGCAGGTATAGTRGGAACATC-3ʹ; *Epiphyas*_COIR: 3ʹ-ATATACTTCAGGRTGACCAAAAAATC-5ʹ), we used the primer design and Oligo primer analysis software Oligo v. 7 (Molecular Biology Insights, Inc. DBA Oligo, Inc., CO, USA) and included reanalyses of published *Epiphyas* spp. COI sequences (i.e., *E. posvittana*, *E. caryotis*, *E*, *dotatana*) to identify conserved 5ʹ and 3ʹ sites for primer design. Primers were optimized for minimal false priming sites, primer duplexes, hairpin loops, and with an optimal PCR annealing temperature of between 52 and 55 °C. PCR amplification of each sample was carried out in a 25 µl total volume and used 0.2 mM of dNTP, 0.2 µM of each forward and reverse primers, 1× MyTaq Reaction Buffer (Bioline), 1.25 units of MyTaq HS DNA Polymerase enzyme (Bioline), and 2.5 µl of the extracted DNA. The PCR profile involved an initial denaturing step at 95 °C for 5 min, 35 cycles of template denaturation/primer annealing/template extension steps for 30 s each at 95 °C/50 °C/72 °C, respectively, and a final template elongation step of 1 cycle at 72 °C for 5 min. All PCR was carried out in a 96-well Applied Biosystems Veriti Thermal Cycler. We visualized PCR products by running 6 µl of each sample with 4 µl of 2× loading dye against 5 µl of 1Kb ladder on 1.5% agarose gels, stained with GelRed and visualized over a UV-transilluminator. For PCR products with no clear amplicon product on the agarose gel, we repeated the PCR by increasing the DNA volume (from 2.5 to 5 µl) and by reducing the water volume accordingly.

The resulting PCR products were purified using Exonuclease I and Antarctic Phosphatase (New England Biolabs, MA, USA) following the manufacturer’s instructions and shipped to Macrogen (Seoul, Korea) for bidirectional sequencing. The mtCOI sequence data were edited and assembled using the PreGap4 and Gap4 programs within the Staden DNA sequence assembly, editing, and analysis package (Staden et al. 2000) to obtain consensus sequence after editing/trimming of poor-quality reads and primer binding sites. For sequence alignment, we used MAFFT v 7.388 (Katoh et al. 2002, Katoh and Standley 2013) in Geneious 11.0.5 (http://www.geneious.com; Kearse et al. 2012) with default parameters (Algorithm: Auto; Scoring matrix: 200PAM / K = 2; Gap open penalty: 1.53; Offset value: 0.123). The consensus sequences were then searched against the NCBI nonredundant database using nucleotide-nucleotide Blast (Altschul et al. 1990) for highly similar sequences (megablast) or the Barcode of Life Database (https://www.boldsystems.org/index.php/IDS_OpenIdEngine). Species identity was considered highly confident when a database match resulted in >97%–100% pairwise mtCOI sequence identity via pairwise nucleotide distance estimates (1-(*p*-dist) × 100%).

### In-Field Inspection

Field inspections were conducted in April 2021 to relate trap catch when apples were mature with the abundance of *Epiphyas* life stages and apple damage detected through visual inspection. Inspections were conducted across 13 apple orchards in which traps were placed in Perth Hills (*n* = 2), Manjimup (*n* = 9), and Pemberton (*n* = 2). Inspections were undertaken within the blocks in which traps were placed or blocks immediately adjoining; a total of 4 blocks were inspected per orchard.

From each block, edges were excluded from random sampling to avoid the edge effect. We then haphazardly selected a row, and within each row, 10 trees were haphazardly selected by zigzagging down the row. From each tree, we meticulously randomly selected 50 leaves from one side of the tree and examined 50 leaves per tree (500 leaves per block) and recorded the presence of *Epiphyas* larvae and of any feeding damage resembling that caused by them. The damage caused by *Epiphyas* larvae is relatively distinctive in Australian apple orchards as they feed within larval shelters created by tying leaves together with silky webs (Suckling and Brockerhoff 2010). And any larvae that resembled *Epiphyas* larvae (if found) were kept to confirm identification.

We randomly selected 50 mature fruits for inspection from the selected apple tree and the 4 trees directly adjacent to it (2 on each side) in the same row (*n* = 250 tree fruits per block). These fruits were thoroughly inspected for the presence of life stages or damages attributable to *Epiphyas* larvae. In cases where fallen fruits were observed, up to 20 of them were selected from beneath 5 trees (*n* = 200 fruits per block) for inspection. Upon identifying surface feeding damage or the presence of silky webs associated with tortricid larvae, the affected fruit or leaf was photographed and then removed for further analysis. In-field inspection occurred on a single occasion per location.

### Statistical Analysis

All trap catch data were converted to moths per trap per week (m/t/w) prior to analysis by dividing trap catch by the time between trap clearances. A nonparametric Levene’s test was then used to verify the equality of variances in the data.

Lure effectiveness (m/t/w) was compared in each locality using a Two-way ANOVA. Lure type was the independent variable, and trap servicing date, and replicate were random factors. Pairwise comparisons were carried out using the Tukey’s test for multiple comparisons between lure types. Where the data did not meet the assumptions of a parametric distribution, an independent sample Mann–Whitney *U*-test was carried out. All analyses were conducted using IBM SPSS statistics version 28.

Relative lure effectiveness was estimated as follows for each targeted moth species in each locality:


$$\begin{array}{l} 1-\frac{\begin{array}{l} \left(Targeted\;moths\;captured\;in\;target\;traps-\;Targeted\;moths\;captured\;in\;nontarget\;traps\right) \end{array}}{Targeted\;moth\;captured\;in\;target\;traps} \end{array}$$

Only moths whose identity was genetically confirmed were included. If a nontarget trap caught the same number of target moths as the target trap, then the relative performance of the nontarget trap to the target trap would be equivalent (100%).

To examine trends in trap catch through the year (from when traps were set up to final trap clearance), average m/t/w in each block ± SD was plotted against time for each sampled year in each of the 3 localities. Based on the results obtained from the genetic identification of trapped moths, we inferred that all moths captured in Perth were *E. postvittana*, while those in Manjimup and Pemberton were *E. pulla*. Only data from target traps were used.

The relationship between moth abundance and fruit infestation rate by trapped species of moths was calculated at the orchard level. Two metrics of moth abundance were used: (i) average m/t/w caught in traps emptied at the time of fruit inspection, and (ii) total moths/trap caught in the season through to the time of fruit inspection. Means and SD were calculated for each of the 3 localities with blocks as replicates.

## Results

### DNA Identification

DNA was extracted from the individual head portion of a total of 474 moths that were captured across the 3 localities during in the lure specificity trial (Table 2). The success of COI barcoding was 96.8%. Only 15 specimens yielded incomplete sequences, which could be attributed to various factors, including handling errors in specimen preservation during DNA extraction steps, among others. The successful DNA sequences were identified at the species level through BLAST analysis and>99% were confirmed by complete match (100% identity) as being either LBAM (279 sequences) or *E. pulla* (189 sequences). Only 3 sequences could not be identified as species (Table 2). Two belonged to other families (Oecophoridae and Geometridae). The third was mostly likely an undescribed *Capua* species (Totricidae).

**Table 2.** Moths caught, their identity based on DNA barcoding, and the relative effectiveness of lures compared to the target lure in the 3 study localities. Means labeled with the same letter do not differ statistically within each orchard (*P* ≥ 0.05). * This unknown species shared 92.8% nucleotide similarity to *Poecilasthena urarcha* (Lep. Geometridae; GenBank accession number: JN273962)

Region	Trap type	Total captured moths	DNA identification					Relative lure effectiveness compared to target lure
			LBAM	*E. pulla*	*E. Sp. (1)*	Other	Failed	
**Perth Hills** **(4 orchards)**	LBAM	260	255^a^	0	–	1*	4	Target lure
	*E. pulla*	19	14^b^	0	–	0	5	5.5%
**Manjimup** **(16 orchards)**	LBAM	10	0	10^a^	0	0	0	19.2%
	*E. pulla*	52	0	52^b^	0	0	0	Target lure
	*E. sp. (1)*	34	0	32^ab^	0	0	2	61.5%
**Pemberton** **(3 orchards)**	LBAM	17	0	17^a^	0	0	0	34.0%
	*E. pulla*	52	0	50^b^	0	2^a^	0	Target lure
	*E. sp. (1)*	30	0	26^ab^	0	0	4	52.0%
** Total**		474	269	187	0	3	15	

^a^These 2 samples included: (i) an individual that shared 92.2% nucleotide similarity to *Barea eclecta* (CCDB-15833-F11; GenBank accession number: KF404515.1), and (ii) an individual with 99% partial mtCOI nucleotide similarity to Capua sp. (ANIC8 voucher 11ANIC-09570; GenBank accession number: KF400096.1).

### Moth Lure Specificity

Only LBAM and *E. pulla* traps were tested in Perth Hills (Table 2). The LBAM trap outperformed the *E. pulla* trap at catching LBAM (*F* = 58.7; *df* = 1; P = 0.001), with the *E. pulla* trap being only 5.5% as effective at catching LBAM as the LBAM traps.

All 3 lures were tested in Manjimup and Pemberton (Table 2). In both localities, *E. pulla* traps outperformed the LBAM trap at catching *E. pulla* (*P*_*Manjumup*_ = 0.004, *P*_*Pemberton*_ = 0.018), with the LBAM trap only being 19% to 34% as effective at catching *E. pulla* moths compared to the *E. pulla* trap. There was no significant difference between the effectiveness of the *Epiphyas sp. (1)* trap at catching *E. pulla* when compared to LBAM and *E. pulla* traps in Pemberton and Manjimup (*P*_*Pemberton*_ = 0.563 _LBAM_ and 0.196 _*E. pulla*_; *P*_*Manjumup*_ = 0.156 _LBAM_ and 0.348 _*E. pulla*_) (Table 2).

### Seasonal Abundance of LBAM and *E. pulla* in Apple Orchards

We used LBAM traps to estimate the seasonal abundance of LBAM in the Perth Hills and *E. pulla* trap for *E. pulla* moths in Manjimup and Pemberton. Although moths were only genetically identified to species in 2021–2022, we assumed that catches in 2020–2021 were representative of the targeted species.

**Fig. 1.**
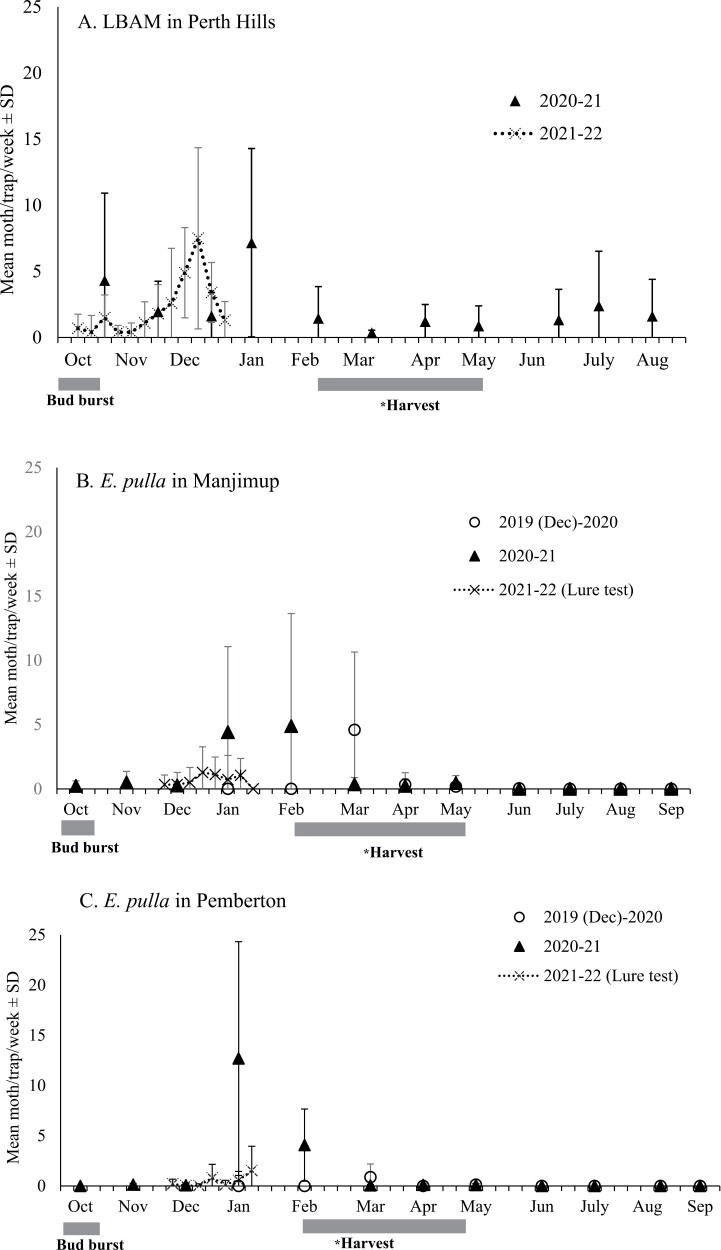
Mean number of *Epiphyas* moths (moths/trap/week) ± SD captured in LBAM traps (*n* = 4) within apple orchards in Perth Hills A) and in *E. pulla* traps (*n* = 16) in Manjimup B) and Pemberton (*n* = 4) C). Traps were set in September with varying clearance intervals between years. *Harvest time varies depending on the apple variety. For example, Pink Lady apples are typically ready for harvest in April, while some other varieties, such as Royal Gala, are harvested in February.

*E. postvittana* densities in the Perth Hills had an initial peak in late October and larger peaks in late December (2020–2021) and January (2021–2022), reaching a mean of approximately 7 moths per trap per week (Fig. 1). The weekly trap clearances in 2021–2022 give a more accurate picture of seasonal trends and show a relatively sharp peak in the second week of December. Average trap catch dropped after January, remaining below an average of approximately 1 moth/trap/week through to the apple harvest (February–May) and into the winter months (Fig. 1A).

Peak *E. pulla* densities reached about 7 moths/trap/week in Manjimup and 13 in Pemberton, but their timing and scale were not consistent between years or localities (Fig. 1). For example, relatively high catches in January and February of 2020–2021 were not observed in 2019–2020.

Trap catch patterns were similar in Manjimup and Pemberton (Fig. 1). Monthly trap clearances in both localities in 2020–2021 suggested either multiple or an extended peak between December and February, after which trap catches were close to zero through to harvest and into winter months. Weekly trapping in 2021–2022 was only conducted through to late January, with mean densities never exceeding 1 moth per trap per week.

### In-Field and Fruit Inspection

A total of 520 trees were inspected across the 3 localities a few weeks prior to harvest. These trees were on 13 commercial orchards on which trapping was being conducted. No evidence of immature stages of *Epiphyas* moths or their damage was observed on either the fruits or the inspected leaves (Table 3). Trapping indicated that moth exposure was quite high across the inspected orchards during that year, although few moths were caught in the month preceding inspections (Table 3).

**Table 3.** Infestation rates were observed in April 2021, along with the corresponding adult male moth catch for the moth species found in each of the 3 localities

	Larval infestation rate estimates			Moth abundance estimates up to when fruit was inspected	
Locality (orchards)	Infestation rate: (*n* = leaves)	Infestation rate: (*n* = fruit on trees)	Infestation rate: (*n* = fruit on ground)	Ave trap catch during harvest (moths/trap/week) ± SD	Season trap catch (moths/trap) ± SD
Perth Hills (n = 2)	0% (*n* = 4000)	0% (*n* = 20,000)	0% (*n* = 1600)	1.00 ± 1.30 LBAM	9.79 ± 16.08 LBAM
Manjimup (n = 9)	0% (*n* = 18,000)	0% (*n* = 90,000)	0% (*n* = 7,200)	1.83 ± 3.88 *E. pulla*	2.07 ± 1.85 *E. pulla*
Pemberton (n = 2)	0% (*n* = 4000)	0% (*n* = 20,000)	0% (*n* = 1,600)	1.41 ± 1.88 *E. pulla*	2.24 ± 1.73 *E. pulla*
Total	***n* = 26,00**0	***n* = 130,00**0	***n* = 10,400**		

## Discussion

This study included 24 commercial apple orchards located across the 3 major apple-growing localities within southwestern Australia. The presence of only 2 of the 3 *Epiphyas* species previously recorded in fruit orchards in that region was validated through DNA sequencing. LBAM was exclusively trapped in Perth Hills, and *E. pulla* exclusively in apple orchards around the towns of Manjimup and Pemberton. *Epiphyas sp. (1)* was not recorded in our study despite the *Epiphyas sp. (1)* and LBAM lures having similar compositions, and *Epiphyas sp. (1)* having previously been caught in low numbers in traps baited with LBAM and *E. pulla* lures (unpublished Department of Agriculture and Food WA (DAFWA) study reported in Hitchcock 2012). Although lures were not species-specific, the LBAM and *E. pulla* lures were effective at monitoring the moth activity of their target species and would, therefore, be best suited for guiding management where only one of the species was known to be present. Both species were most common in apple orchards in summer, becoming relatively rare as harvest season approached. Also, no fruit damage was found at harvest time, suggesting that neither species causes significant losses to mature apples under current commercial management practices.

Limited lure selectivity is not surprising as traps baited with female *E. pulla* adults placed at Roleystone (Perth Hills) only caught male LBAM moths, and traps baited with either female LBAM or *E. pulla* pheromone moths near Manjimup both caught male *E. pulla* moths (Geier and Springett 1976). Many moth species employ multicomponent pheromones for sexual communication. Where sympatric species share common pheromone components, the precise blend composition can become critical for species specificity (El-Sayed et al. 2011). Given the diversity of Australian *Epiphyas* species (Hitchcock 2012) and the presence of E11-14:Ac and E9E11-14:Ac in the pheromone blend of several of them (Horak et al. 1988), ratio differences of the same compounds will help to reduce incompatible mating within this genus. In our study, the 2-component LBAM lure (20:1 ratio of E11-14:Ac and E9,E11-14:Ac) outperformed *E. pulla* lures (10:16 ratio of the same components, together with 2 minor components) when attracting LBAM males. This suggests that the higher ratio of E11-14:Ac is important for catching LBAM, which is consistent with previous observations (Bellas et al. 1983, Muggleston and Foster 1989, Richard Vickers, pers. comm. 2009 cited in Hitchcock 2012).

In many tortricid moth species, minor compounds can elicit peak responses (El-Sayed et al. 2011). The *E. pulla* lure we tested had 2 additional components, 27% of n-14:Ac and 3% of n-16:A. The *E. pulla* lure was more effective than LBAM at attracting *E. pulla* adults, while the 2-component *Epiphyas sp. (1)* lure consistently caught fewer *E. pulla* adults, although it was not statistically significant. Together, this suggests that the substantially higher proportion of E9,E11-14:Ac in the *E. pulla* lure (43% compared to 5% in LBAM and 9% in *Epiphyas sp. (1))* is important. More testing is needed to confirm the role of the additional 2 components that were only in the *E. pulla* lure. However, the composition of the female LBAM sex pheromone gland was reinvestigated using gas chromatographic-electroantennographic detection analysis (GC-EAD) (El-Sayed et al. 2011). Two more components of the female pheromone, (E)-11-tetradecen-l-ol (Ell-I 4OH) and (E)-11-hexadecenyl acetate (Ell-l 6:Ac) were found that elicited electrophysiological responses in the antennae of male *E. postvittana*. When these were added to the standard LBAM lure blend (1% and 0.5%, respectively) in New Zealand trials, trap catches roughly doubled those of traps using the binary blend (El-Sayed et al. 2011, unpublished study referred to in Hitchcock 2012).

In our study, LBAM was exclusively recorded in the Perth Hills, while *E. pulla* was only found in apple orchards around Manjimup and Pemberton. *E. pulla* historically impacted apple orchards in all 3 localities prior to the arrival of LBAM (Geier and Springett 1976, Hitchcock 2012). *Epiphyas postvittana* is thought to have been established at a research apple orchard in Roleystone (Perth Hills) in 1969 following the introduction of seedling stock sourced from an apple orchard in eastern Australia (Geier and Springett 1976). Monitoring on Roleystone found *E. pulla* to be “well represented” in samples in 1970–1971 but absent in the subsequent years, suggesting “possible displacement” by LBAM (Geier and Springett 1976). *E. postvittana* is also thought to have out competed native leafroller species in New Zealand (Wearing et al. 1991). In 2011–2012, a study using LBAM lures in suburban Perth showed moths to be abundant there (Soopaya et al. 2015), but the identity of the moths was not genetically confirmed. However, LBAM has never been recorded in Manjimup or Pemberton localities, including when using traps baited with LBAM males at Manjimup. Further surveys are needed to describe and understand the respective distribution limits of LBAM and *E. pulla*. Specimens would also need genetic identification to confirm their absence and detect hybrids. This can be achieved through techniques such as whole-genome sequencing, as demonstrated in other agriculturally significant arthropod pest species (e.g., Anderson et al. 2016, Elfekih et al. 2021) if they were present (Common 1963, Hitchcock 2012).

Both *E. pulla* and LBAM were most abundant between October and February (late spring and throughout summer) and relatively rare from just prior to, or soon after, the harvest of early harvest varieties commenced. Pink Lady apples are typically ready for harvest in April, while some other varieties, such as Royal Gala, are harvested in February. In Perth Hills, LBAM population peaks in October, corresponding with green flush, through to mid-January when apples had reached the size of golf balls. This phenology corresponded with that observed in suburban Perth in 2011–2012 where trap catches peaked from approximately December to early January and were very low from March through to June (Soopaya et al. 2015). However, peak trap catch was much higher (approximately 100 moths/trap/week) in suburban Perth compared to our study (<10 moths/trap/week). We did not record pest management practices in our surveyed orchards. However, the similarity between the phenology observed in our study and that in suburban Perth suggests the preharvest decline in abundance could at least in part be natural. Parasitism, pathogens, and predation could be a factor, as high levels have been reported on LBAM elsewhere (Geier and Briese 1980a, Danthanarayana 1983, Suckling and Brockerhoff 2010). *Epiphyas postvittana* and *E. pulla* are considered to be polyphagous, and studies on LBAM in ACT (eastern Australia) suggest that the presence of alternative hosts in or adjacent to orchards may also influence population dynamics (Geier and Briese 1980a).

Although both *E. pulla* and LBAM have historically been important pests in apple orchards in southwestern Western Australia, our study suggests that they are not currently causing production losses in commercial orchards. Trap catches were low when apples were mature, and we found no immature moths or apple damage attributable to moths during an extensive survey conducted around harvest time. We did not record pest management practices. However, damage incidence by LBAM in apple orchards in New Zealand has decreased over the past 2 decades following the introduction of pupal and late larval stage-targeting parasitoids from Australia in the 1960s and a shift in fruit production practices from applying broad-spectrum insecticides to selective products (Varela et al. 2010). Parasitoids and predators (especially spiders) have also been observed to cause high mortality in LBAM in Australia (Paull and Austin 2006, Yazdani et al. 2015, Yazdani and Keller 2017). Furthermore, a detailed study on LBAM in an apple orchard near ACT found that the probability of damage to fruits was directly proportional to the number of larvae and of fruits present, indicating that LBAM did not seek out fruits for attack but struck them by chance encounter (Geier and Briese 1980a). In the United States, LBAM is no longer a pest of regulatory significance as it caused less crop damage than anticipated and standard pest management practices for other routine pests have proven effective (Carey et al. 2022).

Our results confirm that *E. pulla* and LBAM lures are effective for monitoring their target species, although genetic identification of trap catch would be necessary if both pests co-occurred. *E. postvittana* (in Perth Hills) and *E. pulla* (in Manjimup and Pemberton) were common in orchards in summer, but no damage was recorded in mature apples in our study, suggesting that they are causing little damage in commercial orchards in Western Australia. This is consistent with New Zealand, where LBAM is now considered to be a minor pest (Harder and Rosendale 2008), and California, where the potential impact of an incursion first detected in 2007 was found to be overstated (Carey et al. 2022). Further work is needed to understand the role of pest management practices and natural enemies in limiting the impact of LBAM and *E. pulla* on apple production. The apparent failure of LBAM to invade Manjimup and Pemberton despite having so rapidly displaced *E. pulla* in Perth Hills in the early 1970s also leaves unanswered questions.
